# A *WHRN* mutation impacts ocular morphology in rhesus macaques

**DOI:** 10.3389/fcell.2026.1818625

**Published:** 2026-07-15

**Authors:** Ana Ripolles-Garcia, Ana Raposo, Sophie M. Le, Jaeho Shim, Sangwan Park, Karolina Roszak, Kira H. Lin, Meher Khan, Sooyoung Lee, Rosie Thienpaitoon, Lillian Gao, Jun Wang, Marguerite F. Knipe, Timothy Stout, Jeffrey Rogers, Rui Chen, Ala Moshiri, Sara M. Thomasy

**Affiliations:** 1 Department of Surgical and Radiological Sciences, School of Veterinary Medicine, University of California, Davis, CA, United States; 2 William R. Pritchard Veterinary Medical Teaching Hospital, School of Veterinary Medicine, University of California, Davis, CA, United States; 3 Department of Ophthalmology and Visual Sciences, Gavin Herbert Eye Institute – Robert M. Branson Center for Translational Vision Research, University of California, Irvine, CA, United States; 4 Department of Ophthalmology, Cullen Eye Institute, Baylor College of Medicine, Houston, TX, United States; 5 Human Genome Sequencing Center and Department of Molecular and Human Genetics, Baylor College of Medicine, Houston, TX, United States; 6 Department of Ophthalmology and Vision Science, School of Medicine, University of California, Davis, CA, United States; 7 California National Primate Research Center, Davis, CA, United States

**Keywords:** ABR, ffERG, OCT, ocular biometry, rhesus macaque, USH2D, Usher syndrome, whirlin

## Abstract

**Background:**

Rhesus macaques are increasingly used to model inherited sensory disorders, yet the phenotypic impact of naturally occurring variants in primate colonies often remains undefined.

**Methods:**

We identified five rhesus macaques homozygous for a missense variant in *WHRN* (p.Val495Met in exon 7), which encodes whirlin and is implicated in human Usher syndrome type 2D, and compared them with five age and sex matched wild-type controls (*WHRN* homozygotes: mean age 9.4 ± 1.8 years; controls: mean age 8.6 ± 1.1 years) using standardized ocular and auditory phenotyping, including comprehensive ophthalmic examination, A-scan ocular biometry, intraocular pressure measurement, cycloplegic refraction, macular optical coherence tomography with retinal layer thickness quantification, full-field electroretinography, and brainstem auditory evoked response testing.

**Results:**

*WHRN* homozygotes showed a consistent shift in ocular component dimensions, with significantly reduced lens thickness, while refractive error remained centered near emmetropia. By contrast, fundus examination and macular optical coherence tomography showed preserved retinal morphology, electroretinography waveforms were comparable between groups, and brainstem auditory evoked responses did not show evidence of overt hearing impairment under the recording conditions used.

**Conclusion:**

These findings define a subtle ocular biometry phenotype associated with *WHRN* p. Val495Met homozygosity in rhesus macaques, while retinal structure and function were preserved at the time of testing; more comprehensive auditory phenotyping across frequencies and thresholds will be needed to assess whether subtle, frequency specific hearing deficits consistent with an atypical Usher presentation are present.

## Introduction

Usher syndrome (USH) is an autosomal recessive, clinically and genetically heterogeneous condition defined by the combination of sensorineural hearing loss and progressive retinal degeneration consistent with retinitis pigmentosa, with vestibular dysfunction present in a subset of affected individuals (Castiglione and Möller, 2022). Many proteins implicated in USH localize to specialized apical structures of cochlear hair cells, particularly the stereocilia, which are actin rich microvilli rather than true cilia, as well as to the photoreceptor connecting cilium; accordingly, USH is widely regarded as a disorder of ciliary associated sensory compartments (Castiglione and Möller, 2022). Epidemiologically, USH has been estimated to account for about 9.2% of congenital profound deafness in children and about 50% of hereditary deafblindness (Castiglione and Möller, 2022; D’Esposito et al., 2025). From the retinal standpoint, USH is the most common syndromic form of retinitis pigmentosa (D’Esposito et al., 2025). Clinically, USH is traditionally grouped into four major subtypes (USH types 1–4) based on the severity and progression of hearing loss, the presence or absence of vestibular involvement, and the typical timing of retinal disease onset (D’Esposito et al., 2025; Velde et al., 2022).

Usher syndrome type 2 (USH2) is the most prevalent subtype, and is typically characterized by congenital bilateral moderate to severe hearing loss that is generally stable over time, with retinitis pigmentosa and vestibular function that is intact or only variably affected (D’Esposito et al., 2025). Visual dysfunction is often diagnosed in adolescence or early adulthood, with an average diagnosis age of ∼24 years for USH2 versus ∼17 years for USH1, reflecting the later onset of retinitis pigmentosa in USH2 (D’Esposito et al., 2025; El-Amraoui and Petit, 2014). Causal genes include *USH2A*, *ADGRV1* (also known as *VLGR1/GPR98*), and *WHRN* (whirlin), which define the USH2A, USH2C, and USH2D subtypes, respectively (D’Esposito et al., 2025; El-Amraoui and Petit, 2014). Although clinical subtypes are defined using hearing severity, vestibular findings, and the relative timing of retinal disease, substantial genetic heterogeneity and genotype phenotype overlap can blur these boundaries, contributing to variability in progression and clinical manifestation across families and even within the same gene (Keats and Savas, 2004). *WHRN* encodes a multi PDZ domain scaffolding protein that helps organize the USH2 protein complex. In the inner ear, it is required for normal stereocilia elongation and architecture, while in photoreceptors it localizes with USH2A and ADGRV1 at the periciliary membrane complex near the connecting cilium, a region critical for intracellular protein transport of photoreceptors (El-Amraoui and Petit, 2014; van Wijk et al., 2006; Holme et al., 2002; Yang et al., 2012). In humans, the canonical long WHRN isoform is a 907 amino acid scaffold protein, and pathogenic variants affecting this retina relevant long isoform are associated with Usher syndrome type 2D, whereas variants affecting the region shared with the short isoform more often cause nonsyndromic hearing loss (Mathur and Yang, 2019; Fuster-García et al., 2021).

To advance Usher syndrome research, there is a critical need for animal models that capture the dual sensory phenotype, with both measurable hearing impairment and retinal disease. In mice, many USH models show clear inner ear phenotypes, yet the retinal phenotype is often absent or mild (Williams, 2008). This limitation has been attributed, at least in part, to species differences in photoreceptor anatomy, including absent or underdeveloped calyceal processes and periciliary membranes in mouse photoreceptors compared with primates, which may reduce vulnerability to disruptions in USH protein networks at the periciliary region (Fuster-García et al., 2021; Williams, 2008). Consistent with the need for primate models, a CRISPR Cas9 generated *MYO7A* (USH1B) gene edited rhesus macaque model was recently reported by the Oregon National Primate Research Center, providing strong proof of concept that non-human primates can reproduce key multisensory features of USH and can serve as translational platforms for therapy development (Ryu et al., 2022).

While this CRISPR-Cas9 gene-edited rhesus macaque model of *MYO7A* (USH1B) has provided a foundation for primate modeling of USH, the field still lacks a complementary primate model for USH2, the most common clinical subtype, and none has been reported for the *WHRN*-associated USH2D form (Ryu et al., 2022). Addressing this gap is important for translational studies because primate retinal architecture and ciliary transport biology more closely resemble the human condition than rodent models. Herein, we report a novel putative deleterious variant in exon 7 of the *WHRN* gene, p. V495M, discovered in five homozygous rhesus macaques (*Macaca mulatta*) at the California National Primate Research Center. This p.Val495Met variant maps to exon 7 of the canonical transcript and lies in the long isoform portion of the protein, close to the transition between the second N terminal repeat and the proline rich region.

We compared ocular parameters in these five mutants with five age- and sex-matched wild-type (WT) controls to evaluate phenotypic similarities with human USH2.

## Methods

### Animals and genetic sequencing

Live examinations of rhesus macaques (*M. mulatta*) were conducted at the California National Primate Research Center (CNPRC), University of California, Davis, an AAALAC International accredited facility. All procedures complied with the Association for Research in Vision and Ophthalmology Statement for the Use of Animals in Ophthalmic and Vision Research and conformed to the National Institutes of Health Guide for the Care and Use of Laboratory Animals. Blood collection, ophthalmic examinations, and phenotyping were performed under a protocol approved by the UC Davis Institutional Animal Care and Use Committee. Venous blood was collected for genomic DNA extraction using standard methods; DNA was stored at −80 °C and subsequently shipped to Baylor College of Medicine (Houston, Texas, USA).

Genetic testing was performed as part of ongoing CNPRC studies aimed at characterizing naturally occurring genetic variation in rhesus macaques, with a focus on identifying variants in inherited retinal disease genes that can support colony management and facilitate genotype informed research studies (Wang et al., 2024). Targeted sequencing, capturing the coding regions of 286 inherited retinal disease genes, and whole exome sequencing were performed in 685 rhesus macaques following the manufacturer protocol. Sequencing reads were aligned to the rhesus reference genome assembly (Mmul_8.0.1 or Mmul_10) using BWA mem, and single nucleotide variants and short insertions and deletions were called using the GATK pipeline. Variants were annotated with Variant Effect Predictor (McLaren et al., 2016) based on merged Ensembl and RefSeq gene models for Mmul_8.0.1 or Mmul_10, and rhesus genome coordinates were lifted over to orthologous human positions. Protein altering effects in human coordinates were annotated using ANNOVAR (v07/17/2017) (Wang et al., 2010) and dbNSFP (v3.5a, including SIFT, PolyPhen 2, and related predictors) (Liu et al., 2016; Liu et al., 2011) based on the hg19 gene model. As described in Wang et al., the missense variant annotation framework also incorporated CADD phred score (v1.6), additional dbNSFP-derived predictors and conservation metrics including REVEL, PolyPhen2_HVAR, phyloP100way, phastCons100way, and GERP, and human population allele frequency data from gnomAD (Wang et al., 2024). Candidate pathogenic variants were identified by screening against the Human Gene Mutation Database.

#### Ophthalmic phenotyping and ocular biometry

Macaques were sedated by intramuscular injection of ketamine hydrochloride (5–30 mg/kg; Ketaset, Zoetis, Parsippany, NJ, USA) and dexmedetomidine (7.5–15 μg/kg; Dexdomitor, Zoetis, Parsippany, NJ, USA), with or without midazolam (0.10 mg/kg; Midazolam Injection, USP, Hikma Pharmaceuticals USA Inc., Columbus, OH, USA). Atipamezole (0.15–0.25 mg/kg, intramuscular; Antisedan, Zoetis, Parsippany, NJ, USA) was administered for reversal of dexmedetomidine sedation following completion of procedures. Body temperature was monitored throughout sedation and actively supported using external warming measures as needed.

Comprehensive ophthalmic examinations were performed by a board-certified veterinary ophthalmologist and included portable slit lamp biomicroscopy, indirect ophthalmoscopy, and streak retinoscopy as previously described (Ross et al., 2026; Lin et al., 2021). Intraocular pressure was measured by a single evaluator using rebound tonometry (TonoVet, Icare, Vantaa, Finland). Pupillary dilation and cycloplegia were induced with topical tropicamide 1% (Akorn Inc., Lake Forest, IL, USA), phenylephrine 2.5% (Paragon BioTeck Inc., Portland, OR, USA), and cyclopentolate 2% (Alcon Laboratories Inc., Fort Worth, TX, USA).

Ocular biometry was acquired using an A-scan ultrasound (PacScan Plus, SonomedEscalon, Lake Success, NY, USA) to measure axial length (AXL), anterior chamber depth (ACD), lens thickness, and vitreous chamber depth (VCD). A 10-MHz A-scan probe was positioned perpendicular to the central cornea with coupling gel (Goniosoft, OcuSoft Inc., Richmond, TX, USA) after topical anesthesia with proparacaine 0.5% (Akorn Pharmaceuticals, Lake Forest, IL, USA). Five acquisitions were obtained using manual freeze when corneal, anterior lens, posterior lens, and retinal echoes were clearly identified with adequate signal amplitude, and the mean value for each parameter was calculated for each eye. Subsequently, B-scan ultrasonography (Butterfly iQ, Butterfly Network, Inc., Guilford, CT, USA) connected to an iPad was used to acquire two-dimensional images of the globe. The probe was placed over the eyelid or directly over the globe with coupling medium, and the small-organ preset was used to obtain images centered on the optic axis.

### Optical coherence tomography (OCT) and retinal layer measurements

Spectral-domain OCT images were acquired using a Spectralis HRA + OCT system (Heidelberg Engineering, Heidelberg, Germany) with a modified chinrest to accommodate the facial contour of rhesus macaques. A 20° × 20° macular volume scan was obtained in high-speed mode (1024 A-scans per B-scan; 25 µm spacing between B-scans), centered on the fovea. In addition, a 30° × 5° raster scan was acquired in high-resolution enhanced-depth imaging mode (1536 A-scans per B-scan; 234 µm spacing between B-scans). Twenty-five frames were averaged for each B-scan using the automatic real-time eye-tracking function. Only scans with a signal strength ≥6 were included. An artificial tear solution (GenTeal; Alcon, Geneva, Switzerland) was applied as needed to maintain corneal hydration during image acquisition.

Retinal and choroidal layer thicknesses were measured manually by the same experienced trained evaluator using the caliper tool in ImageJ (National Institutes of Health, Bethesda, MA, USA). Measured layers included the retinal nerve fiber layer (RNFL), ganglion cell layer (GCL), inner plexiform layer (IPL), inner nuclear layer (INL), outer plexiform layer (OPL), outer nuclear layer (ONL), photoreceptor inner segment (IS), photoreceptor outer segment (OS), retinal pigment epithelium (RPE), choriocapillaris (CC), and outer choroid (OC), along with total retinal thickness (TRT). Measurements were obtained at two parafoveal sites located 1.5 mm nasal and 1.5 mm temporal to the foveal center.

#### Electroretinography (ERG)

Full-field electroretinography (ffERG) was performed after 30 min of dark adaptation using ERG-Jet corneal electrodes and a RETeval system (LKC Technologies, Gaithersburg, MD, USA). Pupils were pharmacologically dilated and topical corneal anesthesia was achieved as described above; corneal lubrication was maintained using a sterile veterinary ocular lubricant gel (Optixcare Eye Lube Plus, Aventix Animal Health, Burlington, ON, Canada). Light stimuli were delivered using the integrated handheld flash stimulator of the RETeval system (LKC Technologies, Gaithersburg, MD, USA) positioned at a fixed working distance from the eye according to manufacturer guidance. Testing followed the standard protocol of the International Society for Clinical Electrophysiology of Vision (ISCEV) and included four dark-adapted recordings: 0.01 cd·s/m^2^ (rod response), 3.0 cd·s/m^2^, 10.0 cd·s/m^2^ (mixed rod and cone response), and oscillatory potentials at 3.0 cd·s/m^2^. After 10 min of light adaptation to a 30 cd/m^2^ background, photopic recordings were obtained at 3.0 cd·s/m^2^ for single-flash responses and 30-Hz flicker (3.0 cd·s/m^2^ at 28.3 Hz). Implicit times (ms) and amplitudes (µV) of the a- and b-waves were recorded, and oscillatory potentials were measured. For the photopic flicker response, the first waveform trough-to-peak was used for analysis. Recordings were captured using manufacturer software, and traces with inadequate signal quality or excessive noise were excluded.

To assess whether a retinal degeneration phenotype could emerge later in life, two *WHRN* homozygous macaques were available for longitudinal re-examination. One macaque was evaluated at 11.33 years and again at 15.67 years, and the second macaque was evaluated at 10.33 years and again at 14.75 years. At both visits, animals underwent macular OCT with retinal layer thickness quantification and ffERG to assess retinal structure and function.

### Auditory Brainstem Response (ABR) testing

Auditory Brainstem Responses (ABR) were recorded using a handheld BAERCOM system (version 1.5, UFI, Morro Bay, CA, USA). Subdermal needle electrodes were placed at the vertex (active electrode), over the right and left mastoid regions (reference electrodes), with the contralateral mastoid functioning as the ground for the recording on each side. Click acoustic stimuli were delivered via insert earphones at an intensity of 80 dBnHL. For each ear, responses were averaged over approximately 1,600–1,800 stimulus presentations (depending on recording quality) to generate a waveform. ABR waveforms were reviewed for the presence and morphology of reproducible peaks and for consistency of interpeak timing across replicate traces.

After the initial cohort assessment, additional auditory testing was performed in the single *WHRN* homozygous macaque available for repeat examination and in three WT macaques for descriptive comparison, using a Duet auditory evoked potential system with SmartEP software (Intelligent Hearing Systems Corp., Miami, FL, USA). Testing included bilateral click ABR threshold assessment and frequency specific tone burst ABR testing at 0.5, 1, 2, 4, and 8 kHz. For each recording, 500 to 1,000 stimuli were averaged at each sound level and tone burst frequency tested. Because only one *WHRN* homozygous macaque was available for repeat testing, these additional data were interpreted descriptively and were not used for statistical comparison.

### Histological evaluation

Histopathologic evaluation was performed in one 14-year-old female rhesus macaque that died for reasons unrelated to the study. The eyes were fixed in 10% neutral buffered formalin and submitted to a diagnostic histopathology laboratory for routine paraffin embedding, sectioning, and hematoxylin and eosin (H&E) staining. Sections from the right and left globes were evaluated for retinal architecture, with particular attention to the retinal ganglion cell layer, inner nuclear layer, outer nuclear layer, and peripheral retinal morphology. The central outer nuclear layer thickness was assessed semiquantitatively by counting the number of nuclear rows. For contextual comparison, slides from similarly aged rhesus macaques were reviewed to estimate the expected range of central outer nuclear layer thickness.

### Statistical analysis

Statistical analyses were performed using GraphPad Prism version 10.6.1 (GraphPad Software, Boston, MA, USA). Differences between groups were assessed using the Mann-Whitney test, and a *P* value < 0.05 was considered statistically significant. To account for multiple comparisons, raw P values from the Mann Whitney tests were adjusted using the Bonferroni method across the different parameters evaluated. Measurements from left and right eyes were averaged to generate a single value per primate for each outcome prior to statistical testing. Because only two macaques had longitudinal follow up, statistical comparisons of change over time were not performed; findings were limited to descriptive characterization and qualitative comparison between timepoints.

## Results

To examine pathogenicity of the *WHRN* p.Val495Met (p.V495M) variant, we used *in silico* prediction and population frequency data from a genomic sequencing database of over 2,000 rhesus macaques reported by Wang et al. (Nature Communications, 2024) (Wang et al., 2024). The variant (rheMac8:15:24986427:G→A; hg38:9:114423457:C→T) is predicted to be damaging by PolyPhen2_HVAR (0.999) and shows additional support for functional impact based on CADD (25.8) and REVEL (0.394) scores, together with strong evolutionary constraint metrics (phyloP100way 7.165, phastCons100way 1.0, and GERP 5.3). These annotations were derived from the Wang et al. framework, which incorporated CADD phred score (v1.6) (Chen et al., 2024), dbNSFP v3.5a derived predictors and conservation metrics, and human population allele frequency data from gnomAD (Wang et al., 2024; Liu et al., 2016; Chen et al., 2024). This variant is located in exon 7 of the major human *WHRN* isoforms (Supplementary Figure S1). The amino acid valine (Val) is conserved across 30 mammals, including 27 primates, with the exception being methionine (Met) at the corresponding position in the reference genome of *Pan paniscus* (Bonobo) (Supplementary Figure S2). This variant does not fall within the currently annotated functional domains according to UniProt (https://www.uniprot.org/uniprotkb/Q9P202/entry). The AlphaFold-predicted structure of the corresponding protein region is shown in Supplementary Figure S3 (Jumper et al., 2021). AlphaMissense classified the variant as ambiguous, with a pathogenicity score of 0.5069 (Cheng et al., 2023). This allele was relatively rare (rhesus allele frequency = 6.824 × 10^−2^) in the reported rhesus macaque cohort, and was extremely rare in the human population (gnomAD v4.1.0 allele frequency = 6.195 × 10^−7^). Collectively, these findings support classification of *WHRN* p.V495M as a variant of uncertain significance (VUS) and suggest that it is a candidate warranting follow-up functional studies using available rhesus macaque models carrying this naturally occurring variant.

Ten rhesus macaques housed at the California National Primate Research Center were included. Five macaques were homozygous for a putative deleterious missense variant in exon 7 of *WHRN*, which encodes whirlin (p.Val495Met, p.V495M) (Wang et al., 2024). The homozygous cohort comprised one male and four females and ranged in age from 6.8 to 11.3 years, with a mean age of 9.4 ± 1.8 years. Five additional macaques were age and sex matched WT controls. The control cohort comprised two males and three females and ranged in age from 6.6 to 9.6 years, with a mean age of 8.6 ± 1.1 years.

### Ophthalmic phenotyping shows biometric changes in *WHRN* homozygotes

Across the homozygous cohort, ophthalmic findings were mild and largely incidental, consisting mainly of subtle lens and fundus changes. In an 11-year-old male, a few small white punctate gliotic spots were noted on the optic nerve head in the right eye, along with perinuclear and posterior cortical lens opacities in both eyes and a mildly posteriorly positioned lens in both eyes. In a 10-year-old female, the ophthalmic examination was completely normal. In one 9-year-old female, punctate pigment was observed on the anterior lens capsule in the right eye with a posterior cortical punctate cataract in the right eye, while in another 9-year-old female, punctate cortical cataracts were present, affecting both the anterior and posterior cortex in the right eye and the posterior cortex in the left eye. In the youngest macaque, a 6-year-old female, punctate pigment was present on the posterior lens capsule in both eyes with extensive small hard drusen at the posterior pole in both eyes. Intraocular pressures were within the normal range for rhesus macaques (8–21 mmHg) (Ollivier et al., 2004; Bito et al., 1979; Tu et al., 2019; Yu et al., 2009), but the *WHRN* homozygotes (14 ± 3 mmHg) were significantly lower than WT controls (18 ± 3 mmHg; *P* = 0.0177; Supplementary Table S1).

**FIGURE 1:**
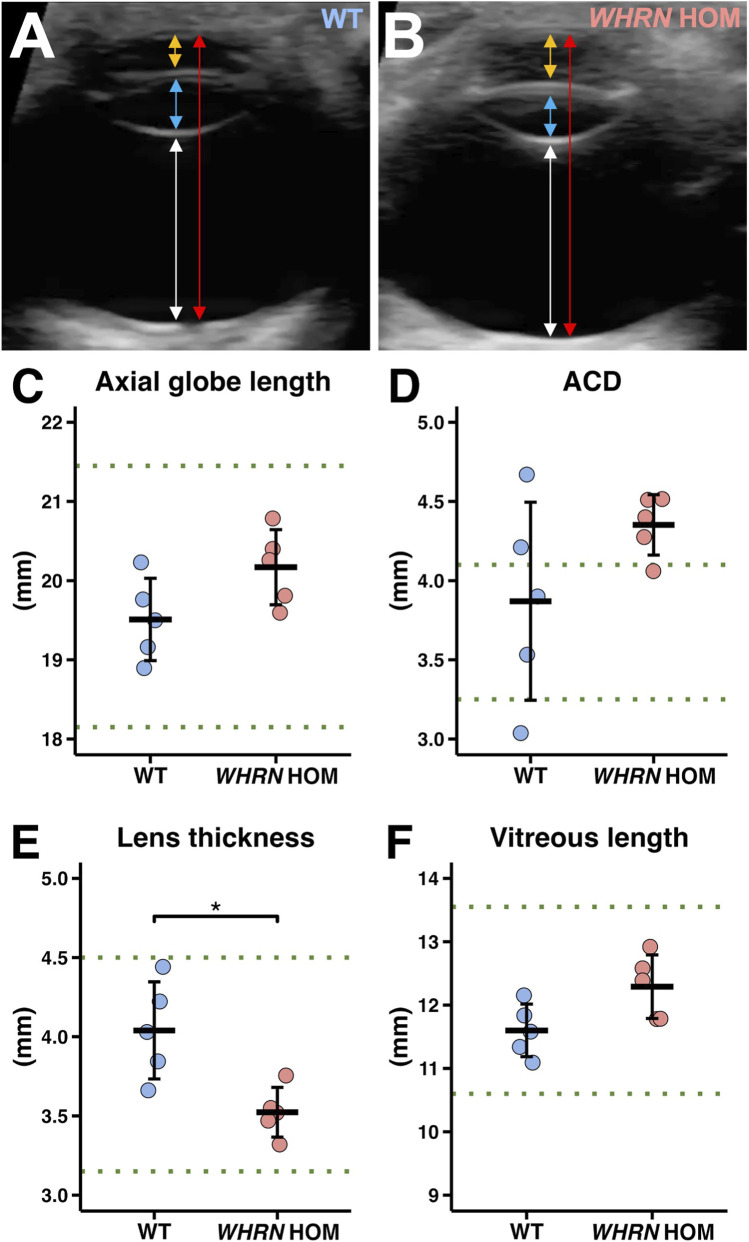
Ocular biometry identifies longer axial globe length and shorter lens thickness in *WHRN* homozygous (HOM) macaques compared with WTs. Representative B mode ocular ultrasound images from the left eye of a WT control (**(A)** male, 11.3 years) and the left eye of a *WHRN* HOM macaque (**(B)** male, 11.8 years) illustrate the axial measurements used for analysis. Measurements include anterior chamber depth (ACD; yellow double headed arrow), lens thickness (blue double headed arrow), vitreous length (white double headed arrow), and axial globe length (red double headed arrow). Quantitative comparisons are shown as box plots for axial globe length **(C)**, ACD **(D)**, lens thickness **(E)**, and vitreous length **(F)** in WT and *WHRN* HOM. Each data point represents the mean value of both eyes for one macaque. Horizontal black lines indicate the group mean, and whiskers indicate the standard deviation. Brackets denote between group comparisons; asterisks indicate statistical significance (*P < 0.05); and dark green dashed lines delineate the reference interval in normal macaques between 5–15 years (Fernandes et al., 2003; IOVS). Differences between groups were assessed using the Mann-Whitney test.

When ocular biometry was compared between *WHRN* homozygotes and WT controls, group differences were detected across multiple ocular components (Figures 1A,B; Supplementary Table S1). Axial globe length was greater in *WHRN* homozygotes (20.17 ± 0.51 mm) than in WT controls (19.51 ± 0.50 mm), although this difference did not remain significant (*P* = 0.2210; Figure 1C). Anterior chamber depth (ACD) did not significantly differ between *WHRN* homozygotes (4.36 ± 0.30 mm) and WT controls (3.87 ± 0.60 mm; *P* = 0.6010; Figure 1D). By contrast, lens thickness was significantly reduced in *WHRN* homozygotes (3.52 ± 0.26 mm) relative to WT controls (4.04 ± 0.30 mm; *P* = 0.0216; Figure 1E). Vitreous chamber length did not significantly differ between *WHRN* homozygotes (12.29 ± 0.50 mm) and WT controls (11.60 ± 0.40 mm; *P* = 0.0947; Figure 1F). Relative to published A-scan ultrasound biometry in adult rhesus macaques aged 5–15 years, median axial globe length (AXL), lens thickness and vitreous chamber depth in *WHRN* homozygotes remained within the expected reference interval, whereas mean ACD exceeded the upper reference limit, indicating a disproportionately deep anterior segment in this cohort (Figure 1, green dashed lines) (Fernandes et al., 2003).

The streak retinoscopy results were within normal limits in the *WHRN* homozygous macaques, as refractive error was mild and centered near emmetropia. Streak retinoscopy identified only mild refractive error overall, centered close to emmetropia across the group (mean spherical equivalent 0.00 ± 0.54 D; median 0.00 D; range −0.75 to +0.75 D; 5 of 10 eyes mildly myopic and 5 of 10 eyes mildly hyperopic). In the oldest macaque (11-year-old male), refractive error was −0.75 D OD and +0.25 D OS. In the next oldest macaque (10-year-old female), refractive error was −0.25 D OD and +0.25 D OS. Among the two 9-year-old females, one had −0.75 D OD and +0.25 D OS, while the other was mildly hyperopic bilaterally (+0.75 D OD and +0.75 D OS). In the youngest macaque (6-year-old female), refractive error was mildly myopic in both eyes (−0.25 D OD and −0.25 D OS).

When refractive error was compared with ocular biometry, there was no evidence of clinically relevant associations in this homozygous cohort. Refractive error remained centered near emmetropia and did not scale with AXL, vitreous chamber depth, lens thickness, or anterior chamber depth. Notably, the eye with the greatest AXL (21.05 mm OS in a 9-year-old female; mean AXL 20.79 mm) was mildly hyperopic (+0.75 D), whereas mild myopia (−0.75 D) occurred in eyes spanning the biometric range, including an eye with a shorter AXL (19.71 mm OD in an 11-year-old male) and an eye with a longer AXL (20.29 mm OD in a 9-year-old female). Overall, these data indicate that refractive status was largely independent of ocular biometry within the narrow refractive range observed. Similarly, refractive error did not show an obvious relationship with ophthalmic examination findings, as mild refractive shifts were observed both in eyes with subtle anterior segment changes (punctate cortical or perinuclear lens opacities, focal pigment on the lens capsule) and in eyes with an otherwise unremarkable examination.

#### Retinal microanatomy is preserved in WHRN homozygous macaques (SD-OCT)

**FIGURE 2:**
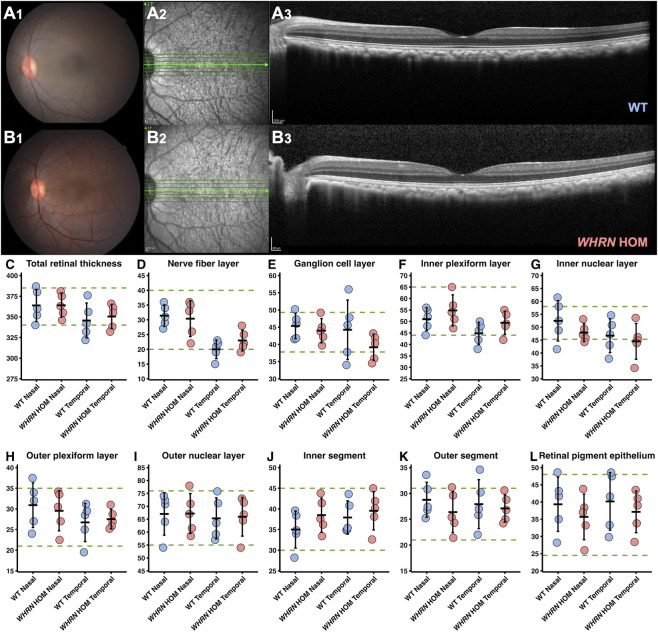
Macular retinal morphology is normal in *WHRN* homozygotes. Color fundus photographs **(A1,B1)** and corresponding infrared reference images **(A2,B2)** are shown for a WT macaque (**(A1–A3)** female, 9.6 years) and a *WHRN* HOM macaque (**(B1–B3)** female, 10 years). Green lines on the infrared images indicate the location of the horizontal macular line scans shown in **(A3,B3)**. Retinal layer thickness measurements obtained from the macular scans at nasal and temporal parafoveal locations are summarized for total retinal thickness **(C)**, retinal nerve fiber layer **(D)**, ganglion cell layer **(E)**, inner plexiform layer **(F)**, inner nuclear layer **(G)**, outer plexiform layer **(H)**, outer nuclear layer **(I)**, photoreceptor inner segment **(J)**, photoreceptor outer segment **(K)**, and retinal pigment epithelium **(L)**. Each data point represents the mean value of both eyes for one animal. Horizontal black lines indicate the group mean, and whiskers indicate the standard deviation. Dark green dashed lines delineate the reference interval in normal macaques (Lin et al., 2021; Yiu et al., 2018). Differences between groups were assessed using the Mann-Whitney test.

Macular SD OCT imaging showed similar retinal morphology in *WHRN* homozygotes compared with WT controls (Figures 2A1–B3). Total retinal thickness was comparable between genotypes at both parafoveal locations (Figure 2C; nasal *P* = 0.6272, temporal *P* = 0.2932). Retinal nerve fiber layer (RNFL) thickness likewise did not differ between groups (Figure 2D; nasal *P* = 0.7792, temporal *P* = 0.0841), and ganglion cell layer (GCL) thickness was also similar (Figure 2E; nasal *P* = 0.4801, temporal *P* = 0.1023). In the inner plexiform layer (IPL), thickness was slightly greater in *WHRN* homozygotes at both the nasal and temporal locations, but neither comparison reach significance (Figure 2F; nasal, *P* = 0.1086; temporal, *P* = 0.2070). Inner nuclear layer (INL) thickness was lower in *WHRN* homozygotes at both locations, although these differences were not significant (Figure 2G; nasal, *P* = 0.4030; temporal, *P* = 0.4071). Outer retinal layers were largely unchanged, including the outer plexiform layer (OPL) (Figure 2H; nasal *P* = 0.9191, temporal *P* = 0.7429) and outer nuclear layer (ONL) (Figure 2I; nasal *P* = 0.8094, temporal *P* = 0.4850). Photoreceptor inner segment thickness was greater nasally in *WHRN* homozygotes, although this difference was not significant (Figure 2J; *P* = 0.1763), and was comparable temporally (*P* = 0.6760). Photoreceptor outer segment thickness was comparable at both locations (Figure 2K; nasal *P* = 0.0831, temporal *P* = 0.5638). Retinal pigment epithelium (RPE) thickness was also comparable between groups (Figure 2L; nasal *P* = 0.2461, temporal *P* = 0.3173). When interpreted against published rhesus macaque reference values (Figures 2C–L, dark green dashed lines), layer thickness distributions generally remained within the expected range for the species (Lin et al., 2021; Yiu et al., 2018).

In the two *WHRN* homozygous macaques that were available for longitudinal follow up, the fundic appearance was unremarkable and repeat macular SD-OCT did not reveal progressive structural change with advancing age (Supplementary Figures S5, S6). Qualitative comparison of matched macular line scans did not show new abnormalities over time, and the overall foveal contour and retinal lamination remained preserved (Supplementary Figure S5). Visualization of retinal layer thickness values across the two timepoints in both animals likewise did not show evidence of progressive thinning any retinal layer, including the outer nuclear layer and photoreceptor segment layers, at either parafoveal location (Supplementary Figure S6).

### Retinal function is maintained in *WHRN* homozygous macaques on ERG testing

**FIGURE 3:**
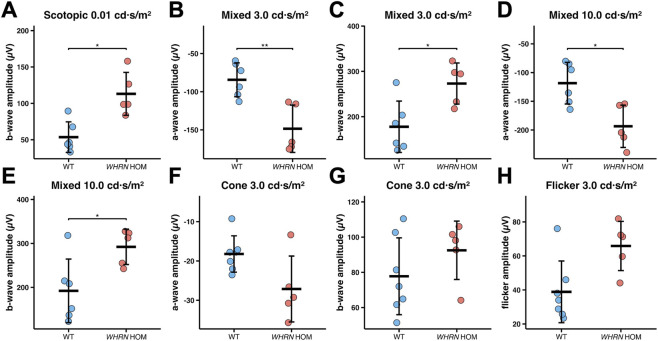
Full-field ERG amplitude measures are normal in *WHRN* homozygous (HOM) macaques. Scatter plots summarize amplitude (µV) parameters from scotopic and photopic full-field ERG recordings in WT (blue) and *WHRN* HOM (red) macaques. Panels show the dark adapted scotopic 0.01 cds/m^2^ b-wave amplitude **(A)**, mixed 3.0 cds/m^2^ a-wave amplitude **(B)** and b-wave amplitude **(C)**, mixed 10.0 cds/m^2^ a-wave amplitude **(D)** and b-wave amplitude **(E)**, light adapted cone 3.0 cds/m^2^ a-wave amplitude **(F)** and b-wave amplitude **(G)**, and 30 Hz flicker amplitude **(H)**. Each dot represents one macaque, with left and right eyes averaged before statistical analysis. Horizontal bars and whiskers indicate mean ± SD. Brackets denote between-group comparisons; significance is annotated above each panel (ns, not significant; *P < 0.05; **P < 0.01). Differences between groups were assessed using the Mann-Whitney test.

When evaluating retinal function by full-field ERG under scotopic conditions (0.01 cd·s/m^2^ light intensity), *WHRN* homozygotes showed a significant delay in the b-wave implicit time (83.83 ± 9.03 ms in WT vs. 94.10 ± 4.38 ms in *WHRN* homozygotes, *P* = 0.0225; Supplementary Figure S7A) together with increased b-wave amplitude (53.58 ± 28.28 µV in WT vs. 113.08 ± 29.00 µV in *WHRN* homozygotes *P* = 0.0137; Figure 3A). Mixed rod-cone 3.0 cd s/m^2^ a-wave implicit time was comparable in both groups (Supplementary Figure S7B) but the a-wave amplitude was larger in magnitude in *WHRN* homozygotes (−84.35 ± 30.84 µV in WT vs. −148.50 ± 30.34 µV in *WHRN* homozygotes, *P* < 0.0081; Figure 3B). The b-wave latency at this same flash intensity was comparable between groups (Supplementary Figure S7C) but the b-wave amplitude was higher in *WHRN* homozygotes (177.74 ± 60.93 µV in WT vs. 273.10 ± 44.26 µV in *WHRN* homozygotes, *P* = 0.0225; Figure 3C). Similar mixed responses were obtained at 10 cd s/m^2^ flash intensity with a comparable a-wave latency (Supplementary Figure S7D) but significantly higher in magnitude a-wave amplitude (−118.47 ± 38.53 µV in WT vs. −193.50 ± 35.82 µV in *WHRN* homozygotes, *P* = 0.0225; Figure 3D). At this light intensity, b-wave latency was delayed in *WHRN* homozygotes (39.39 ± 1.92 ms in WT vs. 45.13 ± 3.20 ms in *WHRN* homozygotes, *P* = 0.0225; Supplementary Figure S7E) and the amplitude was also higher (191.97 ± 71.53 µV in WT bs 292.20 ± 39.00 µV in *WHRN* homozygotes, *P* = 0.0358; Figure 3E).

Under photopic conditions, cone 3.0 cd·s/m^2^ a-wave latency and amplitude were comparable in both groups (Supplementary Figure S7F and Figure 3F). *WHRN* homozygotes had a modest delay in cone 3.0 cd s/m^2^ b-wave implicit time (27.35 ± 1.68 ms in WT vs. 29.36 ± 1.44 ms in *WHRN* homozygotes *P* = 0.0348; Supplementary Figure S7G), with no significant difference in cone 3.0 cd·s/m^2^ b-wave amplitude (Figure 3G). Flicker responses demonstrated shorter implicit time in *WHRN* homozygotes compared with WTs (27.31 ± 1.42 ms in WT vs. 25.47 ± 0.85 ms in *WHRN* homozygotes, *P* = 0.0348; Supplementary Figure S7H) and comparable amplitude (Figure 3H).

**FIGURE 4:**
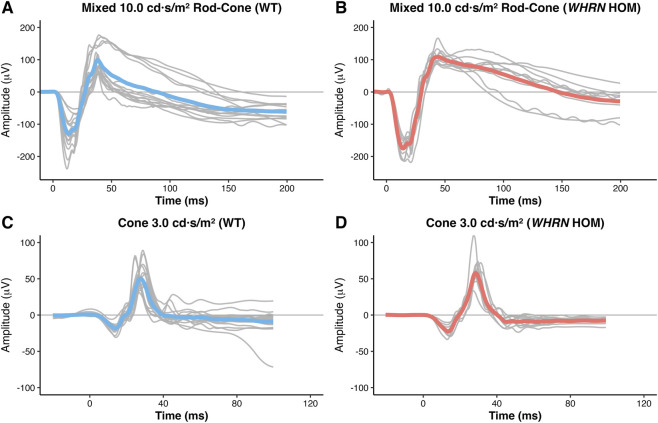
ERG waveforms in *WHRN* mutant rhesus macaques are comparable to WTs. Full-field electroretinography responses are shown for mixed rod/cone recordings **(A,B)** and cone recordings **(C,D)** in wild-type (WT) **(A,C)** and *WHRN* homozygous (HOM) rhesus macaques **(B,D)**. Thin gray traces represent individual eye responses, and the thick colored trace denotes the genotype mean (WT, blue; *WHRN*, coral). Amplitude (µV) is plotted versus time (ms); the horizontal line indicates 0 µV.

When inspecting ffERG waveforms, *WHRN* homozygotes showed preserved overall response morphology compared with WT across both mixed rod–cone and cone-driven recordings (Figures 4A–D). Under dark-adapted mixed rod-cone conditions, the canonical a-wave and b-wave configuration was maintained, with broadly similar waveform timing across the recording window (Figures 4A,B). Likewise, photopic cone responses showed a well-formed cone-mediated a-wave followed by the expected b-wave peak, and overall waveform shape and recovery were comparable between *WHRN* homozygotes and WT (Figures 4C,D). Taken together, these waveform-level findings support preserved global rod-cone and cone pathway function in *WHRN* homozygotes at the ages examined, suggesting that the statistically significant differences detected in select quantitative metrics are unlikely to reflect a clinically meaningful retinal dysfunction.

In the two *WHRN* homozygous macaques with longitudinal data, repeat ffERG recordings remained qualitatively stable over time (Supplementary Figure S8). For the macaque examined at 11.33 years and again at 15.67 years, mixed rod cone and photopic cone responses showed similar waveform morphology, amplitude and timing at both timepoints, without emergence of attenuated b wave amplitudes or delayed implicit times suggestive of progressive retinal dysfunction (Supplementary Figures S8A,B). Likewise, in the macaque examined at 10.33 years and again at 14.75 years, mixed rod cone and cone ERG traces showed comparable amplitudes, implicit times, and overall waveform shape between examinations (Supplementary Figures S8C,D). Because longitudinal ERG data were available for only two animals, statistical comparisons of change over time were not performed and findings are presented as descriptive, qualitative comparisons between timepoints.

### Auditory Brainstem Responses (ABR) in WHRN homozygotes are comparable to WT

**FIGURE 5:**
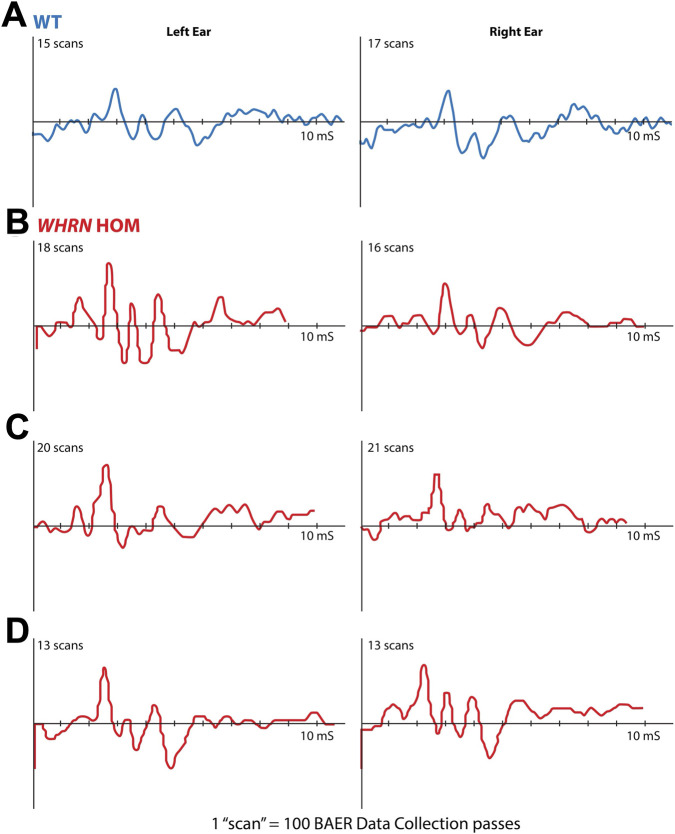
Click Auditory Brainstem Response (ABR) recordings in *WHRN* homozygous macaques were comparable to WTs. Representative ABR waveforms are shown for the left and right ears in four animals tested at 80 dBnHL. **(A)** WT (7.5-year-old female). **(B)** *WHRN* homozygote (7.1-year-old female). **(C)** *WHRN* homozygote (11-year-old female). **(D)** WHRN homozygote (10-year-old female). For each panel, the number of scans collected per ear is indicated; 1 scan = 100 data collection passes, yielding approximately 1,300–2,100 passes per ear across recordings. Traces show reproducible peak morphology and appropriate interpeak timing in representative animals, consistent with normal auditory pathway function. For visualization, ABR waveforms were digitally re-drawn, and the original recordings are provided in Supplementary Figure S1.

The initial click ABR testing demonstrated normal, reproducible waveforms in both ears of the WT control (Figure 5A), with well-defined peaks and consistent interpeak timing between replicate acquisitions, supporting intact brainstem auditory pathway function at the tested stimulus intensity. Similarly, all *WHRN* homozygous macaque exhibited clear, repeatable ABR responses in both the left and right ears (Figures 5B–D), with preserved waveform morphology and appropriate peak timing across traces. Across animals, the number of scan averages acquired per ear supported adequate signal averaging for waveform interpretation.

Additionally, more comprehensive auditory testing was performed in the single *WHRN* homozygous macaque available for repeat examination, with three WT macaques tested for descriptive comparison (Supplementary Figure S9). The retested *WHRN* homozygous macaque showed reproducible click ABR responses and frequency specific tone burst ABR responses across the frequencies evaluated. Response thresholds and waveform morphology were comparable to those observed in WT macaques, with no evidence of overt auditory impairment under the testing conditions used (Supplementary Table S2).

### Histological evaluation

Histopathologic evaluation of both globes from one 14-year-old female *WHRN* homozygous macaque showed preserved ocular and retinal morphology (Supplementary Figure S10). The neuroretina was structurally organized, with preservation of the inner and outer retinal layers. The outer retina was well maintained, including preservation of the outer nuclear layer, photoreceptor inner and outer segment regions, and retinal pigment epithelium. Centrally, the outer nuclear layer was 4–5 nuclei thick, which was comparable to similarly aged rhesus macaques reviewed for comparison, in which the central outer nuclear layer contained 4 to 7 rows of nuclei. Overall, the histologic findings did not support a retinal degeneration phenotype in this animal.

## Discussion

This study also highlights the value of a gene-forward discovery strategy in nonhuman primate colonies (Jaggers et al., 2026). Large scale targeted sequencing and exome datasets can be screened for rare, predicted deleterious variants in genes linked to inherited retinal disease, allowing genotype defined animals to be identified first and then prioritized for focused, prospective phenotyping (Wang et al., 2024; Jaggers et al., 2026). This approach complements the traditional phenotype forward pathway in which ocular or auditory abnormalities prompt genetic investigation, and it is particularly useful when disease expressivity is subtle, variable, or age-dependent. Indeed, using this approach, a valuable rhesus macaque model of autosomal dominant optic atrophy was identified by first detecting a pathogenic *OPA1* p. Ala8Ser variant in the large sequencing cohort and then prioritizing variant carriers for targeted ocular phenotyping (Wang et al., 2024; Jaggers et al., 2026). In the present work, recognition of a homozygous *WHRN* p. Val495Met allele in the CNPRC population enabled assembly of a small, genetically defined cohort and rapid cross-sectional evaluation of ocular structure, retinal function, and auditory pathway responses. Importantly, this strategy supports more efficient use of animals by focusing procedures on a small, genetically defined subset rather than broadly screening large numbers of animals. Even with limited data, establishing this genotype to phenotype baseline provides a foundation for longitudinal follow up, for expanding the cohort as additional carriers are identified, and for testing whether this allele behaves as hypomorphic or produces later onset disease in nonhuman primates.

Systematic phenotyping of naturally occurring genetic variants in rhesus macaques is essential to define genotype-phenotype relationships and to expand the pipeline of clinically relevant large animal models for inherited ocular disease (Wang et al., 2024; Moshiri et al., 2019). Rhesus macaques are excellent models for inherited sensorineural diseases due to their anatomical and genetic similarities to humans with multiple analogous conditions identified (Seah et al., 2022). Relevant animal models of genetic conditions such as USH are paramount, particularly since there are no currently available treatments for the irreversible blindness and deafness that results from this condition; therefore, development and evaluation of novel therapeutic strategies remains a major unmet need (Grotz et al., 2022; Sun et al., 2016). In this context, the identification of five rhesus macaques homozygous for a *WHRN* p. Val495Met variant provides an opportunity to rigorously evaluate a candidate model for USH2D. Therefore, we performed thorough ocular and auditory phenotyping of *WHRN* p. Val495Met homozygous rhesus macaques, providing foundational data to assess the suitability of this naturally occurring genotype as a large animal model for USH2D.

When we evaluated ocular biometry in *WHRN* homozygous macaques compared with WT controls, we identified a distinct pattern of biometric changes. We observed that lens thickness was significantly reduced compared with WT, supporting a contribution of lenticular anatomy. Despite longer AXL dimensions, refractive error remained near emmetropia, consistent with optical compensation in which reduced lenticular power and or altered effective lens position offsets the expected myopic shift from axial elongation (Han et al., 2022). Because retinal development can influence global ocular growth, these biometric changes could arise from retina driven remodeling with secondary lens adjustments that preserve refractive error near zero (Wallman et al., 1987; Smith et al., 2009; Mutti et al., 2005). Collectively, these findings define a subtle biometric phenotype that warrants further mechanistic study and an extended longitudinal assessment. In patients, USH2D due to *WHRN* mutations is typically characterized by normal early ocular development with later onset retinal degeneration and congenital, moderate, stable hearing impairment (Whatley et al., 2020), and published clinical series do not describe abnormal ocular biometry, although these studies were not designed to evaluate biometric outcomes (Sun et al., 2016; Colombo et al., 2015; Herrera et al., 2008). The mild ocular phenotype observed here may therefore reflect an early or subclinical stage, preceding overt retinal degeneration or vision loss.

Similarly, retinal layer measurements were largely preserved in *WHRN* homozygous macaques. Macular OCT showed intact lamination and comparable retinal layer thickness to controls, with only subtle regional variations that remained within published normative ranges (Lin et al., 2021). This aligns with prior work in other animal models, showing that many *Whrn*/USH2 rodent models do not develop robust retinal dystrophy, with degeneration reported only at advanced ages in *Whrn* knockout mice (∼28 months) and limited photoreceptor loss across most USH2 mouse models (Yang et al., 2012; Delmaghani and El-Amraoui, 2022). Full field electroretinography likewise showed no evidence of clinically meaningful rod or cone pathway dysfunction at the ages examined; although several parameters differed from WT, they shifted opposite to degeneration, with larger scotopic a- and b-wave amplitudes across multiple flash intensities and increased photopic flicker amplitudes, alongside only modest implicit time changes in select conditions. Using a commonly applied rhesus macaque to human aging ratio of approximately 1:3, the ages examined here correspond roughly to human mid-adulthood, approximately 44–47 years (Xue et al., 2023). Given that retinitis pigmentosa in USH2D typically begins in late adolescence or early adulthood (Yang et al., 2012), the absence of detectable retinal disease at these approximate ages argues against a classical retinal phenotype associated with *WHRN* p.Val495Met homozygosity in this cohort (Mathur and Yang, 2015). Taken together, these findings suggest that the ages examined were sufficient to rule out a classic retinal degeneration phenotype associated with *WHRN* p. Val495Met homozygosity in rhesus macaques. Nevertheless, assessment at later life stages remains warranted to determine whether a mild, atypical, or non-classical phenotype could develop with aging. Histopathology of both globes in one homozygous macaque confirmed preserved retinal architecture with no evidence of retinal degeneration.

In this study, *WHRN* homozygous macaques showed ABR waveforms at 80 dB that were broadly comparable to the WT control, supporting preserved auditory pathway function under the recording conditions used. Additional audiologic phenotyping was subsequently performed in a single *WHRN* homozygous macaque available for repeat examination and in three WT macaques for descriptive comparison. The *WHRN* homozygous macaque showed click and tone burst thresholds that were broadly comparable to those of WT macaques across the frequencies evaluated, providing no evidence of overt threshold level or high frequency auditory impairment under the expanded testing conditions. However, because only one *WHRN* homozygous macaque was available for repeat auditory testing, these findings should be interpreted descriptively and cannot exclude subtle, age dependent, or incompletely penetrant auditory deficits in the broader genotype. This limitation is particularly relevant because USH2-associated hearing loss in humans is typically high-frequency predominant and generally stable (Castiglione and Möller, 2022). *Whrn* mutant mouse lines show prominent auditory phenotypes ranging from profound hearing loss to more moderate ABR threshold elevations, depending on the allele (Mathur and Yang, 2015). Together, these findings suggest that p. Val495Met does not produce a readily detectable auditory phenotype in the single retested macaque, but expanded audiologic phenotyping across larger cohorts and later life stages will be required to define auditory expressivity in this primate genotype.

Clinically, *WHRN* variants produce either a syndromic USH2D or an isolated hearing-loss phenotype (DFNB31) without retinal disease. This divergence appears largely isoform dependent, reflecting differential disruption of long versus short whirlin isoforms (Mathur et al., 2015; Mburu et al., 2003; Ebermann et al., 2007). Whirlin has long and short isoforms with distinct expression and function in the retina and inner ear, and prior work supports that variants predicted to disrupt the long isoform, are more likely to be associated with Usher syndrome type 2D, whereas variants preferentially affecting short-isoform function more often present as DFNB31 non-syndromic hearing loss (Mathur et al., 2015; Yang et al., 2010). Notably, the p. Val495Met variant characterized here lies in exon 7 of the canonical protein-coding *WHRN* transcript (WHRN-201) and a coding region included in the long whirlin isoform in patients, indicating that this genotype has the potential to affect retina-relevant *WHRN* function; however, to our knowledge, the exact orthologous amino acid substitution has not been reported (Mathur et al., 2015). Because *WHRN* is highly conserved across primates and whirlin performs similar roles in the cochlea and photoreceptor ciliary compartments, the rhesus macaque provides a strong translational context; however, species-specific differences in splicing and genetic background may modulate phenotypic expressivity and these findings need to be interpreted with caution.

The *WHRN* p. Val495Met variant evaluated here represents a rare, naturally occurring missense change in rhesus macaques; to date, this precise amino acid substitution has not been described as a disease-causing allele in the human or animal *WHRN* literature that originally established *WHRN* as an USH2D/DFNB31 gene. Consistent with the broader challenge of modeling USH2 retinal disease in rodents, multiple *Whrn* mouse alleles show prominent inner ear involvement with absent, mild, or delayed retinal manifestations (El-Amraoui and Petit, 2014; Mathur et al., 2015). The classic spontaneous whirler allele (*Whrn* ^wi/wi^), which deletes exons 6 to 9, causes congenital profound hearing loss with vestibular dysfunction but has not been associated with overt retinal degeneration even at advanced ages (Mathur et al., 2015; Mburu et al., 2003; Yang et al., 2010). By contrast, targeted disruption of the long whirlin isoform in *Whrn* knockout mice (deletion at the 5 prime end of exon 1) yields a congenital severe auditory phenotype together with a slowly developing retinal phenotype that becomes detectable only late in life, with reduced ERG amplitudes and photoreceptor layer thinning reported at approximately 28–33 months (Yang et al., 2010). A complementary gene trap line (*Whrn* ^*tm1a/tm1a*^), an engineered allele that inserts a gene-trap cassette into the *Whrn* locus to prematurely terminate transcription and markedly reduce whirlin expression, similarly produces profound hearing impairment with late onset photoreceptor loss; importantly, rescue using bacterial artificial chromosome (BAC) transgenesis (delivery of a large genomic DNA construct carrying the gene with regulatory elements) in whirler mice indicates that restoring the WHRN long isoform is necessary to recover normal retinal function (Mathur et al., 2015). Together, these mouse studies reinforce that *Whrn* related retinal disease can be difficult to elicit in rodents, is strongly allele and isoform dependent, and may emerge only after prolonged aging, which provides important context for the absence of a clear retinal phenotype in the *WHRN* p.Val495Met macaques at the ages examined.

In summary, systematic ocular and auditory phenotyping of *WHRN* p. Val495Met homozygous rhesus macaques identified a subtle but consistent ocular biometry and anterior segment phenotype, while macular structure and ffERG responses remained preserved at the ages examined, and ABR screening did not reveal overt abnormalities under the recording conditions used. These findings highlight the value of deep phenotyping of naturally occurring primate variants to refine genotype-phenotype relationships and to build a pipeline of clinically relevant large animal models for inherited sensory diseases. This cohort provides a well-defined foundation for expanded, age stratified retinal, lenticular, and frequency specific auditory assessments to determine whether additional manifestations emerge over time. Notably, even in the absence of a robust retinal or auditory disease phenotype, this comprehensive dataset is highly informative for variant annotation and may aid future clinical interpretation of *WHRN* p. Val495Met in diagnostic settings.

## Data Availability

The raw data supporting the conclusions of this article will be made available by the authors, without undue reservation.
