# Supplementary material for: A WHRN mutation impacts ocular morphology in rhesus macaques
Source: Front Cell Dev Biol. 2026 Jul 15;14:1818625. doi: 10.3389/fcell.2026.1818625 (PMC13415932; doi:10.3389/fcell.2026.1818625)
Supplement: Supplementary file 1 [file Supplementaryfile1.pdf]

A *WHRN* mutation impacts ocular morphology in rhesus macaques

Ana Ripolles-Garcia[1], Ana Raposo[1], Sophie M. Le[1], Jaeho Shim[1], Sangwan Park[1], Karolina Roszak[1], Kira H. Lin[2], Meher Khan[1], Sooyoung Lee[1], Rosie Thienpaitoon[1], Lillian Gao[1], Jun Wang[3], Marguerite F. Knipe[1], Timothy Stout[4], Jeffrey Rogers[5], Rui Chen[3], Ala Moshiri[6], Sara M. Thomasy[1,6, 7]

Address correspondence to: Sara M. Thomasy, Department of Surgical and Radiological Sciences, School of Veterinary Medicine, Department of Ophthalmology & Vision Science, School of Medicine, University of California at Davis, 1 Shields Avenue, 1220 Tupper Hall, Davis, California 95616, USA. Phone: 530.752.1770; Email: smthomasy@ucdavis.edu

Affiliations:

[1]Department of Surgical and Radiological Sciences, School of Veterinary Medicine, University of California, Davis, CA, USA. [2]William R. Pritchard Veterinary Medical Teaching Hospital, School of Veterinary Medicine, University of California, Davis, CA, USA. [3]Department of Ophthalmology and Visual Sciences, Gavin Herbert Eye Institute – Robert M. Branson Center for Translational Vision Research, University of California, Irvine, CA, USA. [4]Department of Ophthalmology, Cullen Eye Institute, Baylor College of Medicine, Houston, TX, USA. [5]Human Genome Sequencing Center and Department of Molecular and Human Genetics, Baylor College of Medicine, Houston, TX, USA. [6]Department of Ophthalmology & Vision Science, School of Medicine, University of California, Davis, CA, USA. [7]California National Primate Research Center, Davis, CA, USA.

Supplementary Figures

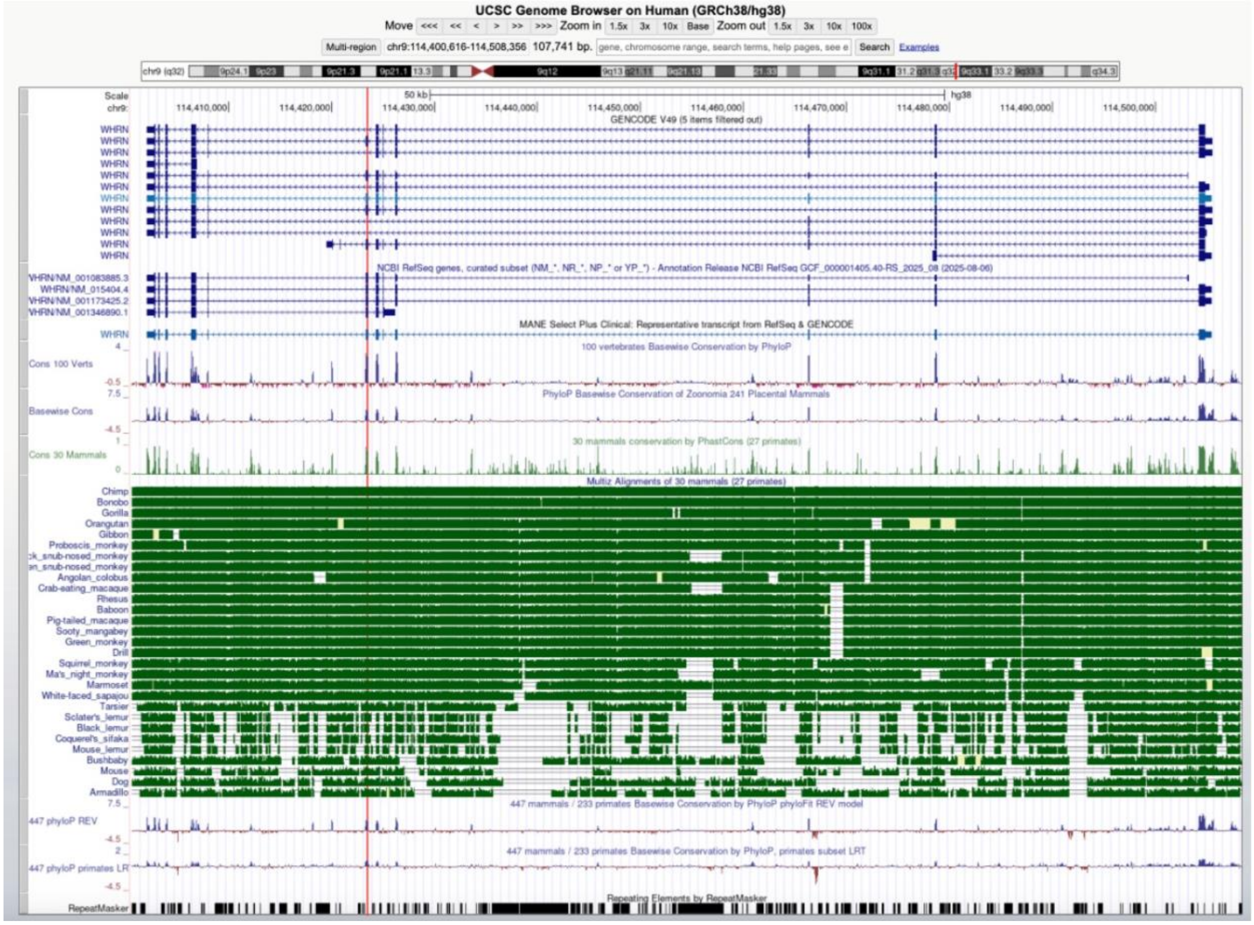

Supplementary Figure S1. Genomic location of the *WHRN* p.Val495Met (p.V495M) variant in human *WHRN* isoforms as visualized in the UCSC Genome Browser. The variant is located within exon 7 of the canonical *WHRN* transcript and is present in coding regions shared across multiple isoforms, including the long whirlin isoform implicated in Usher syndrome type 2D. Gene models, transcript isoforms, and conservation tracks are shown, highlighting the position of the variant within a conserved coding region.

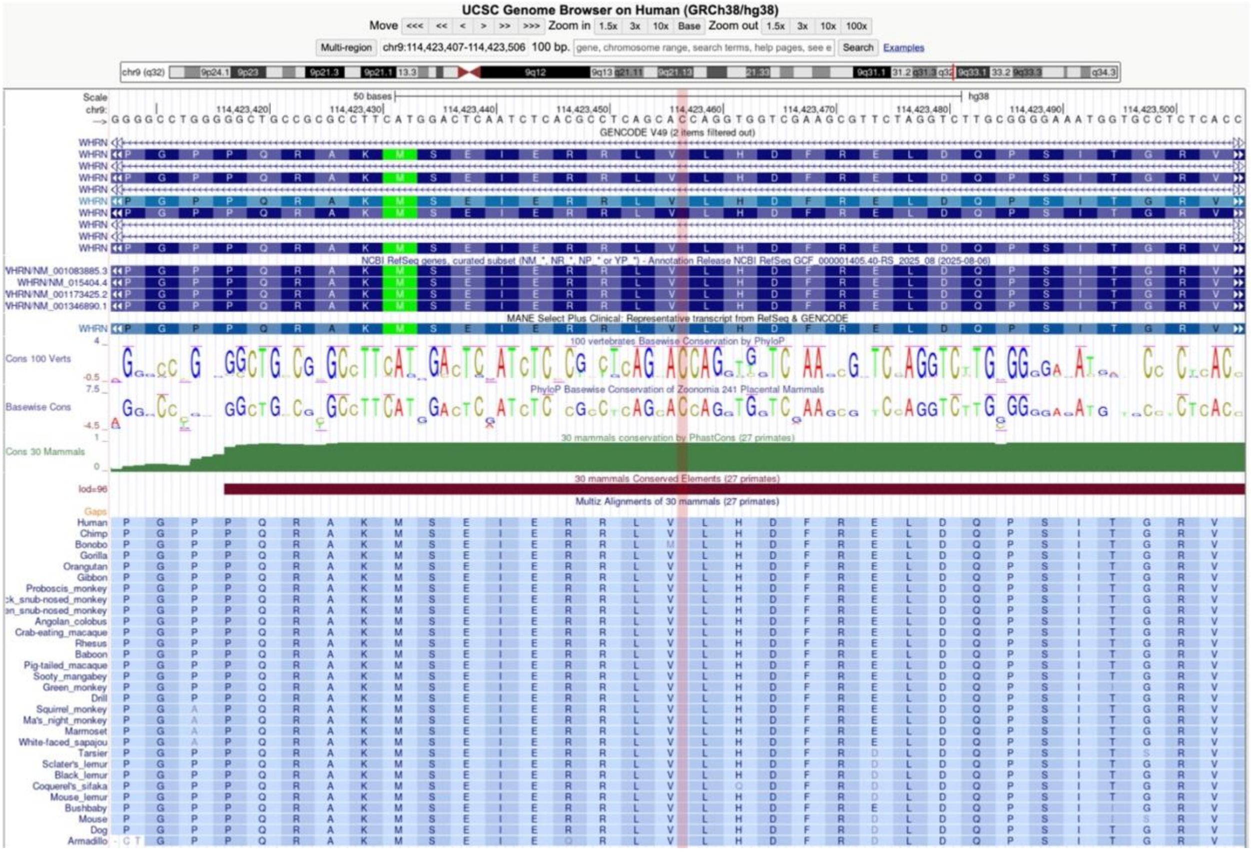

Supplementary Figure S2. The conservation of the amino acid at the position 495 of the *WHRN* isoforms across 30 mammals (including 27 primates) as visualized in the UCSC Genome Browser. Multiple sequence alignment demonstrates that this residue is highly conserved across 30 mammals, including 27 primate species. Conservation tracks and amino acid alignments highlight strong evolutionary constraint at this position, supporting potential functional relevance of the p.Val495Met (p.V495M) variant.

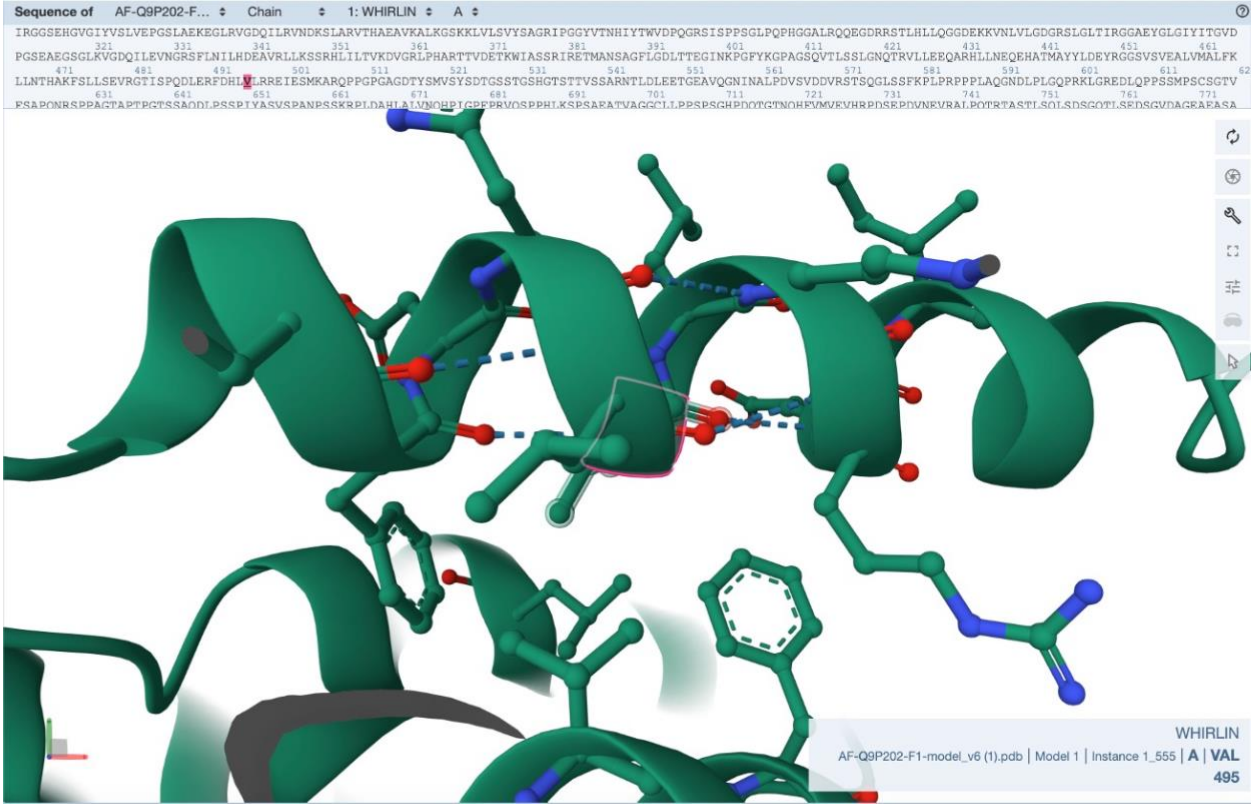

Supplementary Figure S3. AlphaFold-predicted structure of the WHRN protein region surrounding p.Val495, visualized using the RCSB PDB 3D Viewer (https://www.rcsb.org/3d-view). The image highlights the local secondary structure and spatial environment of residue Val495 within the WHRN protein, providing structural context for the p.Val495Met (p.V495M) substitution.

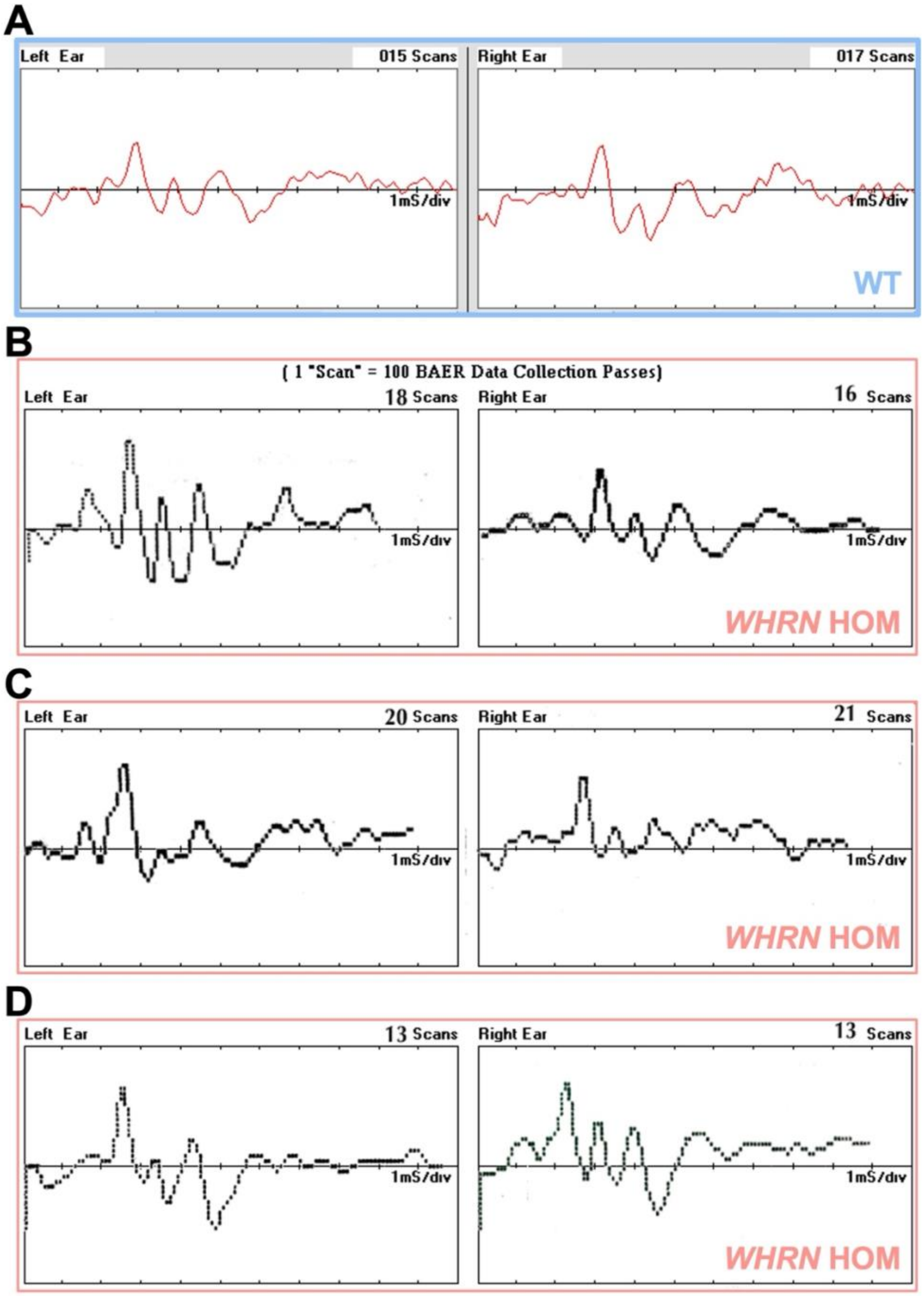

Supplementary Figure S4. Auditory Brainstem Response (ABR) recordings in *WHRN* mutant homozygotes macaques were comparable to WT. Representative ABR waveforms are shown for the left and right ears in four animals tested at 80 dBnHL. **A**, WT (7.5-year-old female). **B**, *WHRN* homozygote (7.1-year-old female). **C**, *WHRN* homozygote (11-year-old female). **D**, *WHRN* homozygote (10-year-old female). For each panel, the number of scans collected per ear is indicated; 1 scan = 100 data collection passes, yielding approximately 1,300-2,100 passes per ear across recordings. Traces show reproducible peak morphology and appropriate interpeak timing in representative animals, consistent with normal auditory pathway function.

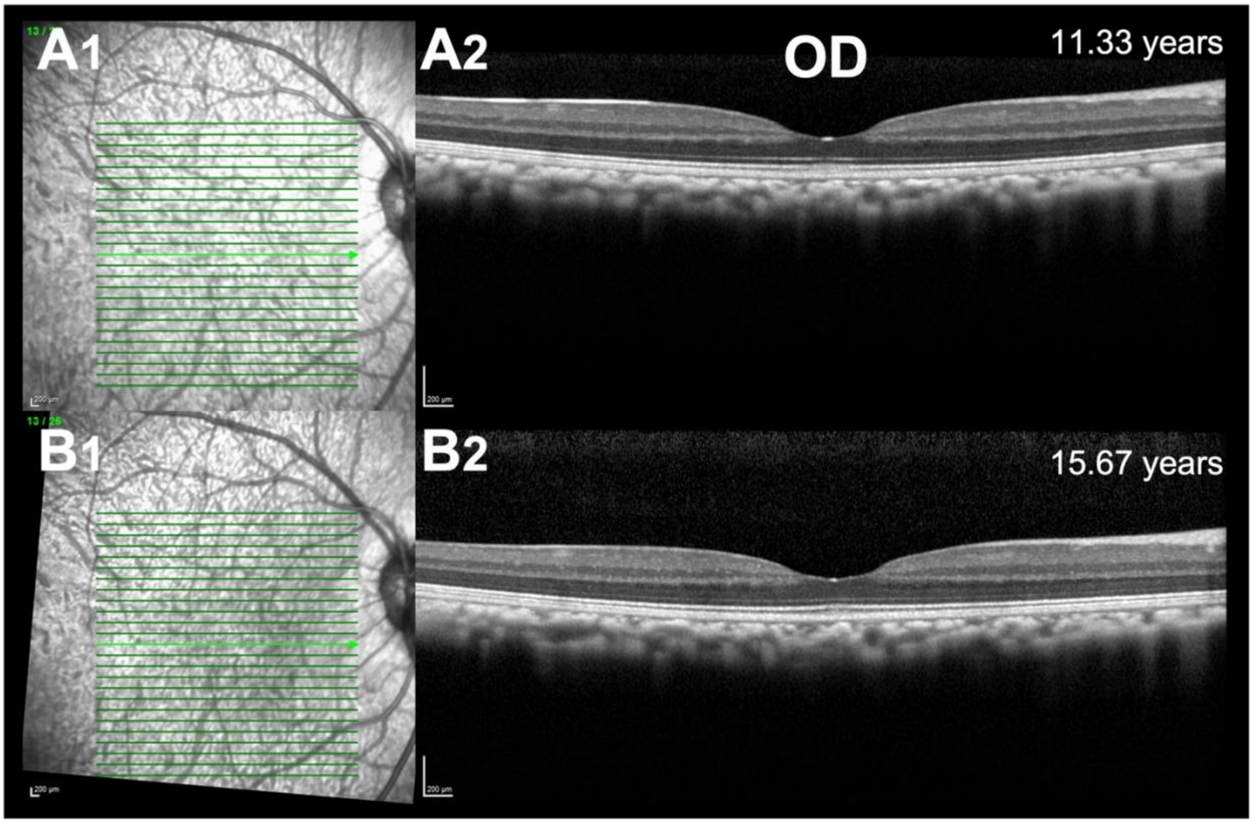

Supplementary Figure S5. Longitudinal macular SD-OCT in a representative *WHRN* homozygous rhesus macaque demonstrates preserved macular structure over time with retinal lamination and overall foveal contour comparable between examinations. Infrared fundus image with scan location (**A1**) and corresponding horizontal macular B scan (**A2**) from the right eye (OD) obtained at 11.33 years. Repeat imaging from the same eye at 15.67 years showing the same scan position (**B1**) and preserved macular microanatomy on the corresponding B scan (**B2**).

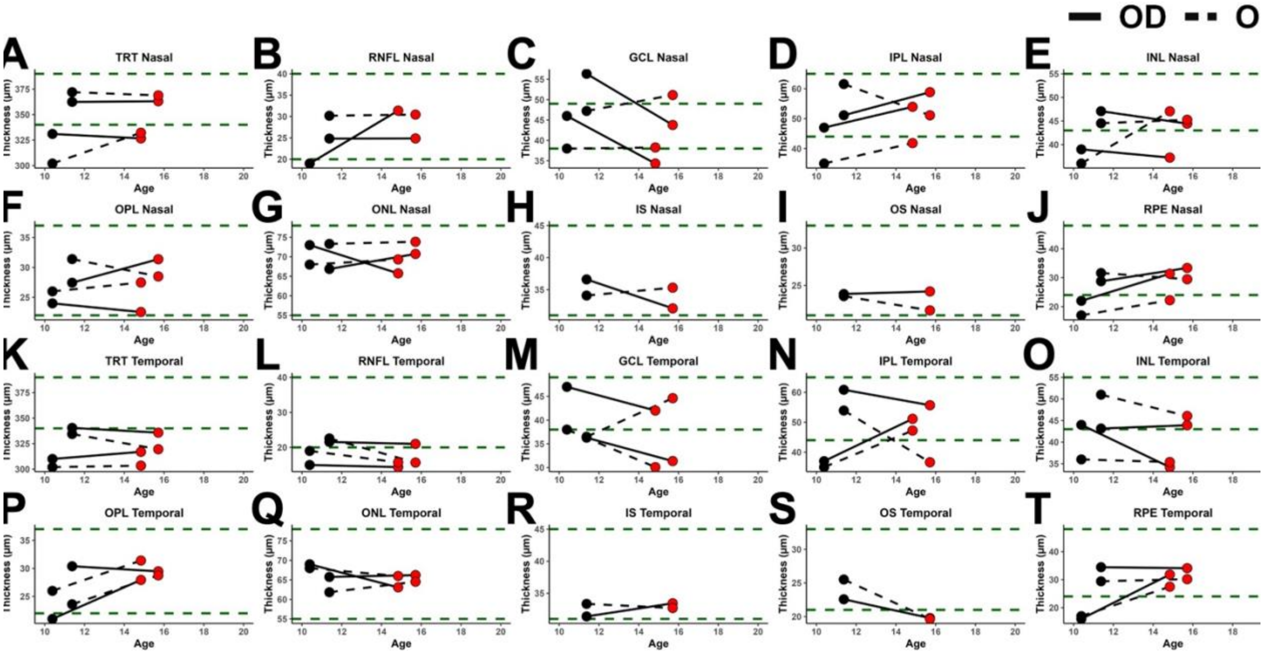

Supplementary Figure S6. Longitudinal macular retinal layer thickness in two *WHRN* homozygous rhesus macaques demonstrates no evidence of progressive thinning of the retinal layers over time. Retinal layer thickness (μm) is shown for total retinal thickness (TRT), retinal nerve fiber layer (RNFL), ganglion cell layer (GCL), inner plexiform layer (IPL), inner nuclear layer (INL), outer plexiform layer (OPL), outer nuclear layer (ONL), inner segment (IS), outer segment (OS), and retinal pigment epithelium (RPE) measured at the parafoveal nasal (**A-J**) and temporal (**K-T**) locations (1.5 mm from the foveal center). Black points indicate retinal measurements at the initial examination (11.33-year-old for one macaque and 10.33-year-old for the other), and red points indicate follow up (15.67-year-old and 14.75-year-old, respectively). Solid traces indicate the right eye (OD) and dashed traces indicate the left eye (OS), with lines connecting the two timepoints for each eye. Dark green dashed lines indicate published rhesus macaque reference limits for each retinal layer (Yiu *et al.* 2018, Exp Eye Res; Lin et al. 2021, TVST) (16,22).

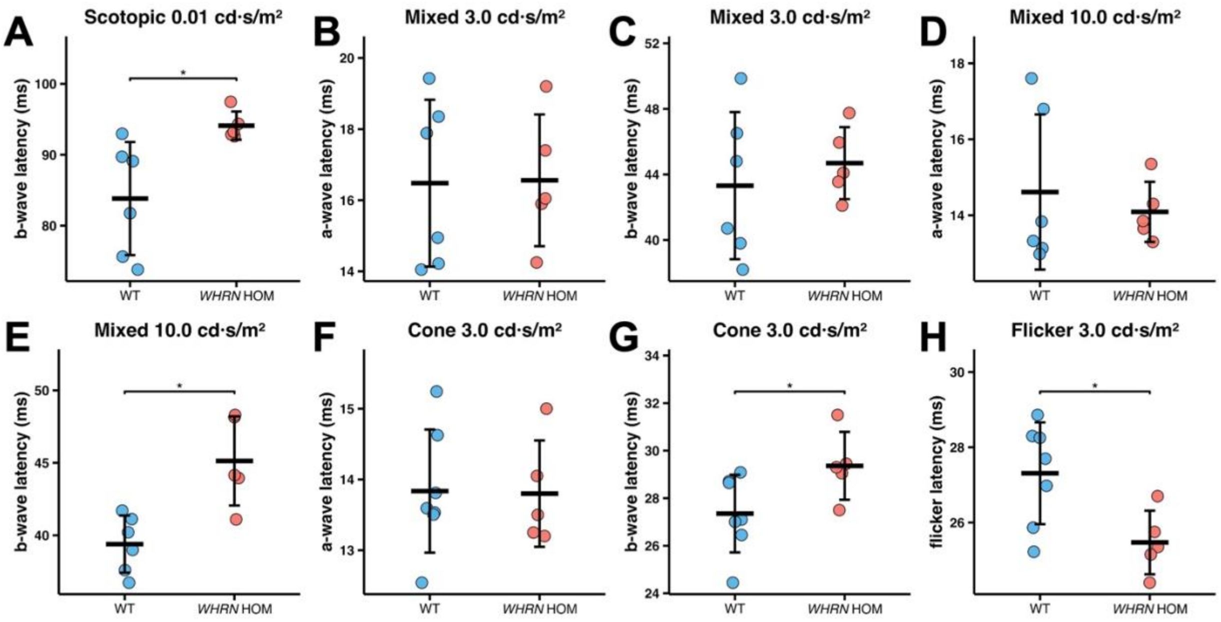

Supplementary Figure S7. Full-field ERG implicit time (latency) measures are normal in *WHRN* homozygous (HOM) macaques. Scatter plots summarize latency parameters (ms) from scotopic and photopic full field ERG recordings in WT (blue) and *WHRN* HOM (red) macaques. Panels show the dark adapted scotopic 0.01 cd·s/m² b-wave latency (**A**), mixed 3.0 cd·s/m² a-wave latency (**B**) and b-wave latency (**C**), mixed 10.0 cd·s/m² a-wave latency (**D**) and b-wave latency (**E**), light adapted cone 3.0 cd·s/m² a-wave latency (**F**) and b-wave latency (**G**), and 30 Hz flicker latency (**H**). Each dot represents one macaque, with left and right eyes averaged before statistical analysis. Horizontal bars and whiskers indicate mean ± SD. Brackets denote between-group comparisons, and significance is annotated where present (* $P<0.05$). Differences between groups were assessed using the Mann-Whitney test.

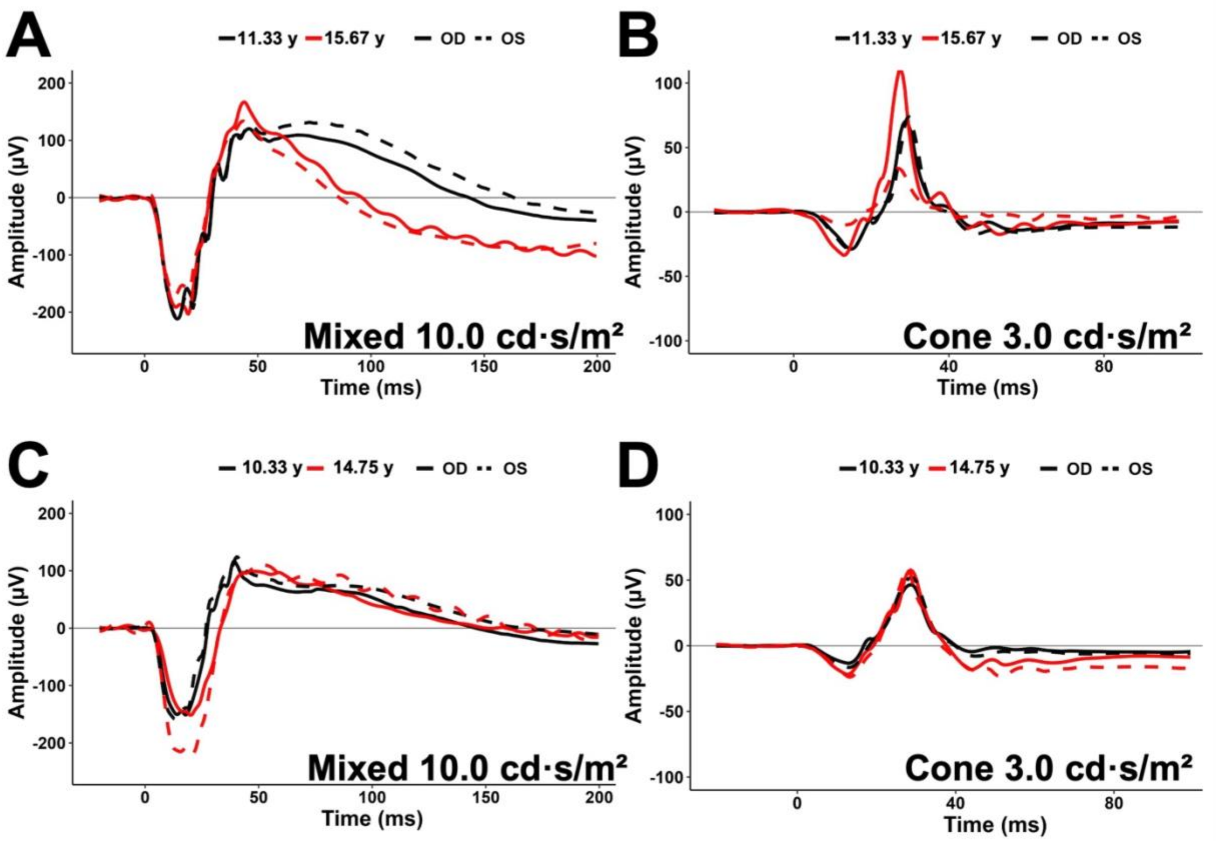

Supplementary Figure S8. Longitudinal full field electroretinography in two *WHRN* homozygous rhesus macaques shows preserved waveform morphology and comparable timing across timepoints. Mixed rod cone response (**A**) and photopic cone response (**B**) from one macaque examined at 11.33 years of age (black) and again at 15.67 years of age (red). Mixed rod cone response (**C**) and photopic cone response (**D**) from a second macaque examined at 10.33 years (black) and again at 14.75 years (red). Solid traces indicate the right eye (OD) and dashed traces indicate the left eye (OS).

FIGURE HERE – NEW ABR

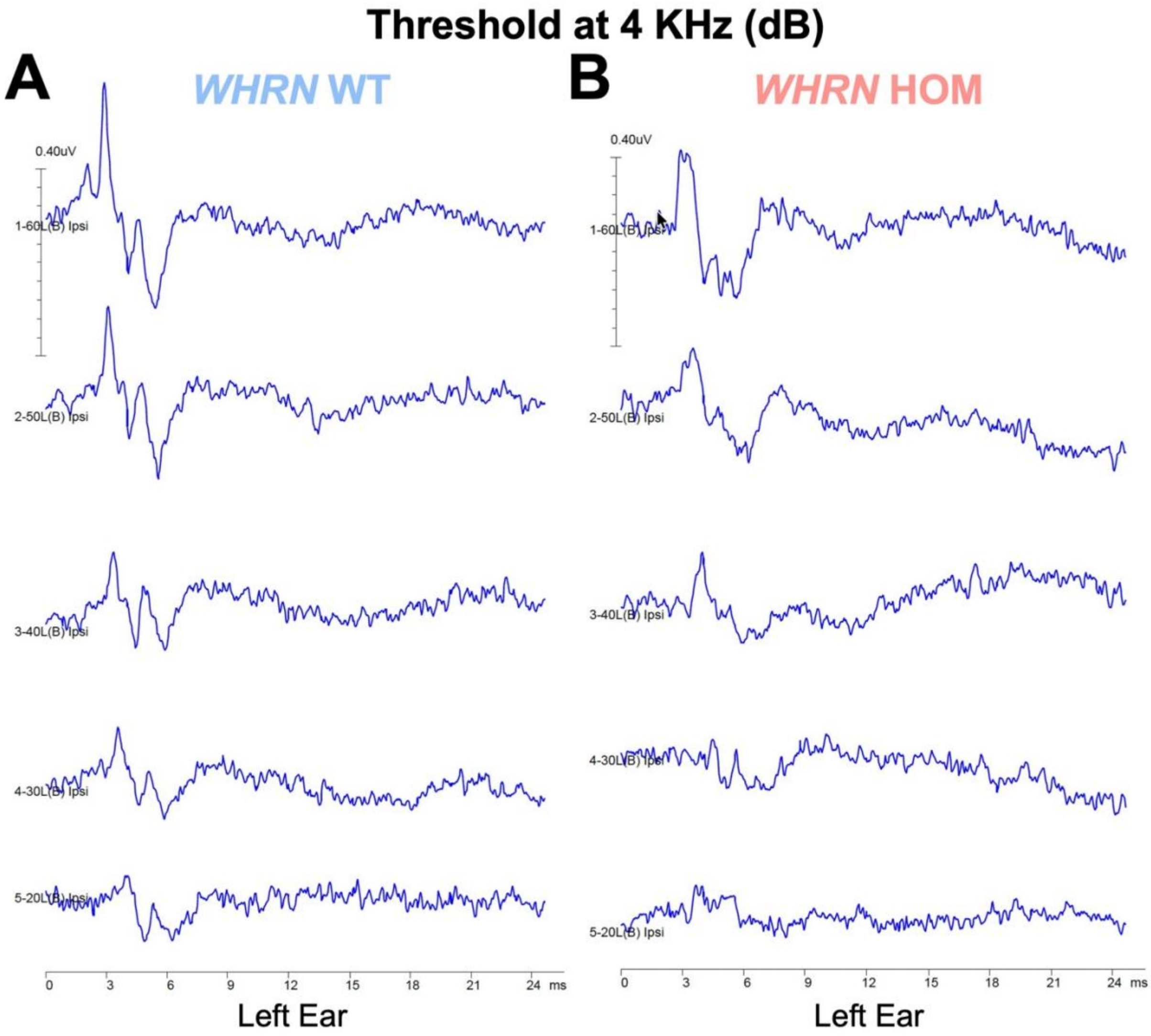

Supplementary Figure S9. Frequency specific Auditory Brainstem Response (ABR) recordings at 4 kHz in a *WHRN* homozygous macaque were comparable to WT. Representative 4 kHz tone burst ABR threshold waveforms are shown for the left ear of two animals tested at decreasing stimulus intensities. **A**, WT (14-year-old male). **B**, *WHRN* homozygote (15-year-old female). Traces are shown across decreasing stimulus intensities from 60 to 20 dB. Waveforms demonstrate reproducible peak morphology at higher intensities, with loss of identifiable responses at lower intensities, supporting a similar 4 kHz auditory response profile in the *WHRN* homozygous macaque and WT animal.

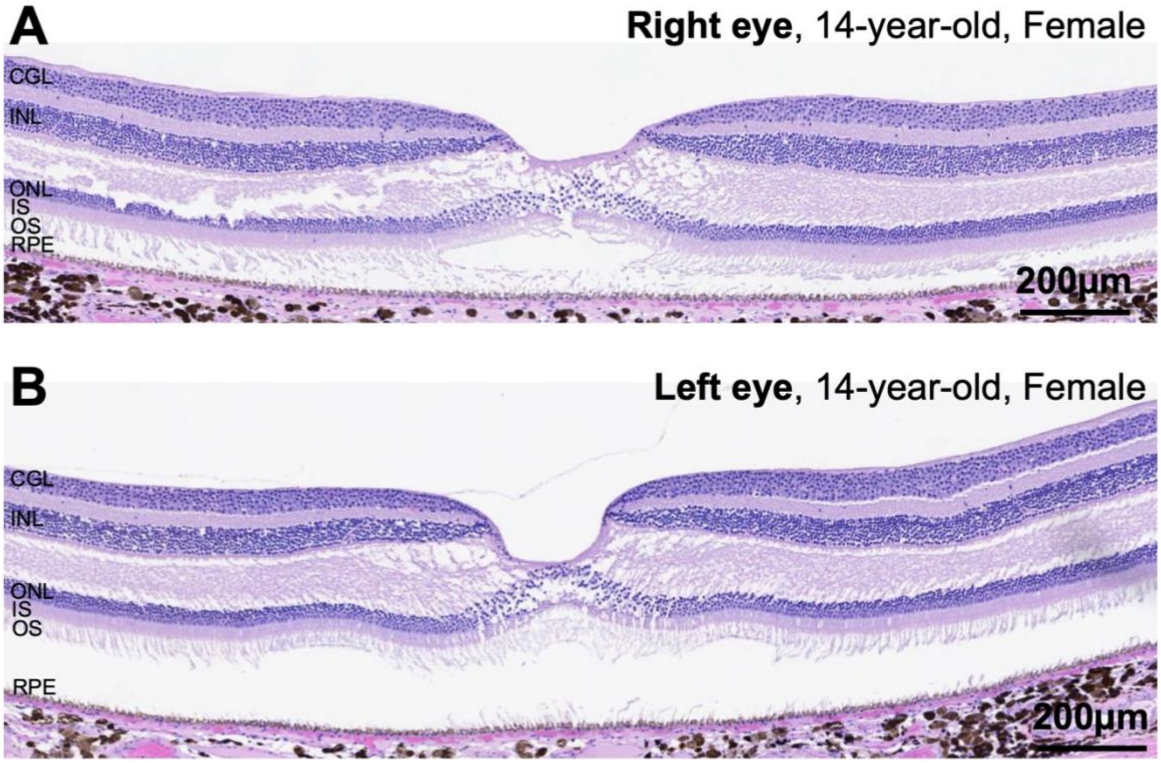

Supplementary Figure S10. Histologic evaluation of both globes demonstrates preserved retinal architecture in a 14-year-old female *WHRN* homozygous rhesus macaque. A, right eye from a 14-year-old female *WHRN* homozygous macaque. The section is centered on the foveal region and illustrates preserved retinal architecture, with normal organization of the inner and outer retina. The outer nuclear layer (ONL), photoreceptor inner and outer segment region, and retinal pigment epithelium are maintained. **B**, left eye from the same animal showing similar findings, with preserved lamination of the inner and outer retinal layers and no histologic evidence of retinal degeneration. The focal retinal separation is considered artifactual, as portions of the photoreceptor outer segments remain attached to the retinal pigment epithelium. GCL, ganglion cell layer; INL, inner nuclear layer; ONL, outer nuclear layer; IS, photoreceptor inner segments; OS, photoreceptor outer segments; RPE, retinal pigment epithelium. Scale bars = 200 µm.

Supplementary Tables

Supplementary Table S1. Individual eye intraocular pressure measurements and ocular biometry in *WHRN* homozygous and wild-type rhesus macaques.

Macaque ID	Eye	IOP	Axial globe length	Anterior chamber depth	Lens thickness	Vitreous length
**HOM 1**	OD	12	20.51	4.68	3.25	12.58
	OS	11	20.01	4.12	3.69	12.2
**HOM 2**	OD	17	19.25	3.73	4.11	11.42
	OS	18	19.94	4.39	3.4	12.15
**HOM 3**	OD	17	20.52	4.32	3.48	12.73
	OS	18	21.05	4.7	3.16	13.11
**HOM 4**	OD	11	20.29	4.34	3.47	12.49
	OS	12	20.51	4.21	3.63	12.67
**HOM 5**	OD	11	19.71	4.44	3.54	11.73
	OS	10	19.91	4.59	3.58	11.83
**WT 1**	OD	20	19.69	4.13	4.13	11.79
	OS	19	19.84	4.29	4.32	11.88
**WT 2**	OD	17	19.04	3.47	3.80	11.26
	OS	18	19.28	3.60	3.89	11.42
**WT 3**	OD	20	20.16	4.54	4.33	12.06
	OS	21	20.30	4.80	4.55	12.25
**WT 4**	OD	18	19.42	3.80	3.99	11.52
	OS	19	19.58	4.00	4.07	11.64
**WT 5**	OD	11	18.80	2.90	3.60	11.00
	OS	16	18.99	3.17	3.72	11.18

Note: HOM indicates homozygous *WHRN* p.Val495Met macaques; WT, wild-type macaques; OD, right eye; OS, left eye.

Supplementary Table S2. Click and frequency specific tone burst auditory brainstem response (ABR) thresholds in one *WHRN* homozygous macaque and three WT macaques.

Genotype	Sex	Age	Ear	Click ABR threshold (dB)	Tone burst threshold at 0.5 KHz (dB)	Tone burst threshold at 1 KHz (dB)	Tone burst threshold at 2 KHz (dB)	Tone burst threshold at 4 KHz (dB)	Tone burst threshold at 8 KHz (dB)
WHRN Homozygous	Female	15y	Right Ear	40 dB	60 dB	60 dB	40 dB	20 dB	50 dB
			Left Ear	50 dB	40 dB	50 dB	50 dB	20 dB	50 dB
WT	Female	18y	Right Ear	50 dB	50 dB	50 dB	30 dB	30 dB	50 dB
			Left Ear	50 dB	50 dB	50 dB	40 dB	30 dB	50 dB
WT	Male	14y	Right Ear	40 dB	60 dB	50 dB	40 dB	20 dB	40 dB
			Left Ear	40 dB	50 dB	40 dB	30 dB	20 dB	40 dB
WT	Female	16y	Right Ear	50 dB	40 dB	50 dB	40 dB	30 dB	50 dB
			Left Ear	60 dB	40 dB	40 dB	30 dB	30 dB	50 dB

Note: ABR, auditory brainstem response; WT, wild type; WHRN, whirlin. Threshold values represent the lowest stimulus intensity, in dB, at which a reproducible ABR waveform was identified for each ear. Recordings were obtained using a Duet auditory evoked potential system with SmartEP software (Intelligent Hearing Systems Corp., Miami, FL, USA). For tone burst testing, frequency specific thresholds were assessed at 0.5, 1, 2, 4, and 8 kHz, with 500 to 1000 stimuli averaged for each sound level at each frequency. Because only one *WHRN* homozygous macaque was available for repeat testing, these data are presented descriptively and were not used for statistical comparison.
